# Irradiation specifically sensitises solid tumour cell lines to TRAIL mediated apoptosis

**DOI:** 10.1186/1471-2407-5-5

**Published:** 2005-01-14

**Authors:** Patrizia Marini, Angelika Schmid, Verena Jendrossek, Heidrun Faltin, Peter T Daniel, Wilfried Budach, Claus Belka

**Affiliations:** 1 Department of Radiation Oncology, University of Tübingen, Experimental Radiation Oncology, Hoppe-Seyler-Str. 3, D-72076 Tübingen, Germany; 2 Clinical and Molecular Oncology, University Medical Center Charité, Lindenberger Weg 80, D-13125 Berlin-Buch, Germany; 3 Department of Radiotherapy and Radiation Oncology, Moorenstr. 5, D-40225 Düsseldorf, Germany

## Abstract

**Background:**

TRAIL (tumor necrosis factor related apoptosis inducing ligand) is an apoptosis inducing ligand with high specificity for malignant cell systems. Combined treatment modalities using TRAIL and cytotoxic drugs revealed highly additive effects in different tumour cell lines. Little is known about the efficacy and underlying mechanistic effects of a combined therapy using TRAIL and ionising radiation in solid tumour cell systems. Additionally, little is known about the effect of TRAIL combined with radiation on normal tissues.

**Methods:**

Tumour cell systems derived from breast- (MDA MB231), lung- (NCI H460) colorectal- (Colo 205, HCT-15) and head and neck cancer (FaDu, SCC-4) were treated with a combination of TRAIL and irradiation using two different time schedules. Normal tissue cultures from breast, prostate, renal and bronchial epithelia, small muscle cells, endothelial cells, hepatocytes and fibroblasts were tested accordingly. Apoptosis was determined by fluorescence microscopy and western blot determination of PARP processing. Upregulation of death receptors was quantified by flow cytometry.

**Results:**

The combined treatment of TRAIL with irradiation strongly increased apoptosis induction in all treated tumour cell lines compared to treatment with TRAIL or irradiation alone. The synergistic effect was most prominent after sequential application of TRAIL after irradiation. Upregulation of TRAIL receptor DR5 after irradiation was observed in four of six tumour cell lines but did not correlate to tumour cell sensitisation to TRAIL. TRAIL did not show toxicity in normal tissue cell systems. In addition, pre-irradiation did not sensitise all nine tested human normal tissue cell cultures to TRAIL.

**Conclusions:**

Based on the in vitro data, TRAIL represents a very promising candidate for combination with radiotherapy. Sequential application of ionising radiation followed by TRAIL is associated with an synergistic induction of cell death in a large panel of solid tumour cell lines. However, TRAIL receptor upregulation may not be the sole mechanism by which sensitation to TRAIL after irradiation is induced.

## Background

TRAIL (Tumour necrosis factor related apoptosis inducing ligand) is one of the most promising anti-cancer agent being currently under investigation (for review see [1-5]). Initially it was shown that TRAIL specifically induces tumour cell apoptosis and tumour regression in nude mice, even when applied as single agent [6,7]. In the meantime rare reports questioned the tumour cell specificity of TRAIL since it was shown that TRAIL induced apoptosis also occurred in normal human liver cells [8,9]. However, subsequentially it was shown that the biochemical preparation of TRAIL rather than TRAIL itself was responsible for the observed toxic effect on hepatocytes [10]. This is strongly supported by the lack of toxicity of TRAIL receptor agonists in humans in first phase I studies [11].

One possible explanation for the tumour specifity of TRAIL could lie in its potential role as a mediator of tumour immune surveillance *in vivo*. In this regard it has been shown that mice lacking TRAIL display a significantly reduced capacity to eliminate syngenic tumour cells in the liver [12,13].

Although TRAIL is a member of the death receptor ligand family certain differences exist to the well characterised ligands TNF (Tumour necrosis factor) or CD95. Most important in this regard is the fact that at least five, instead of only two resp. one, different TRAIL receptors have been identified: The proapoptotic DR4/Trail-R1 [14] and DR5/TRAIL-R2/TRICK2 [15-18] as well as TRAIL-R3/DcR1/TRID [16,33,20] and TRAIL-R4/DcR2/TRUNDD [21,22], which lack any pro-apoptotic function. The latter were shown to protect cells from TRAIL induced apoptosis by competing with the agonistic receptors for TRAIL binding. In addition, TRAIL-R4/DcR2 is able to induce NF-κB activation, which might upregulate a wide array of anti-apoptotic proteins [19-21,23,24]. The role of the fifth TRAIL binding protein, the soluble osteoprotegerin (OPG) is still unclear, since it displays only low binding affinity to TRAIL at physiological temperatures [25,26].

In analogy with the signalling events triggered by CD95, multimerisation of the agonistic TRAIL receptors induces the recruitment of the FADD adapter molecule to the receptor, leading to a subsequent autoproteolytic activation of initiator caspase-8 [27,28]. Active caspase-8 in turn triggers the proteolytic activation of downstream caspases including caspase-3. Downstream caspases ultimately degrade a broad range of cellular proteins and apoptosis is finalized (for review see [29]).

Up to now, caspase-8 was shown to be the most crucial mediator of TRAIL induced apoptosis [30,31]. However, it has been shown that caspase-10 may act as a surrogate for caspase-8 in some cell systems [32,33].

In contrast to receptor mediated apoptosis, DNA damage triggers apoptosis mainly via mitochondrial death pathways (for review see [34,35]). Key step of mitochondrial apoptosis pathways is the mitochondrial release of pro-apoptotic mediators including cytochrome c. This release is generally controlled by a complex interplay of pro-apoptotic members of the Bcl-2 family namely Bax, Bak, Noxa and Puma. Activation of either of those molecules may occur directly via conformational changes [36] or transcriptional upregulation [37-39]. Cytochrome c released from the mitochondria triggering the activation of caspase-9 by association with APAF-1 in an ATP dependent manner [39,40]. Caspase-9 subsequently activates the downstream effector caspase cascade including caspase-3 and, in analogy to receptor mediated apoptosis, cell death is finalised.

With only very few exceptions [41], apoptosis induction via mitochondrial death pathways is abrogated by anti-apoptotic members of the Bcl-2 family. Anti-apoptotic proteins of the Bcl-2 family interfere with the cytochrome c release from mitochondria on multiple stages [42-44].

Interestingly, both (death receptor mediated and mitochondrial) pathways are interconnected on several levels. In case of death receptor activation, the propagation of the apoptotic signal is enhanced by caspase-8 mediated activation of Bid [46]. Bid like other BH3-only molecules triggers the release of cytochrome c from mitochondria ultimately resulting in activation of caspase-9 [45,40]. Thus, receptor mediated death pathways are directly connected to mitochondrial death pathways. Vice versa, activation of caspase-9 via the mitochondrial pathway results in secondary activation of caspase-8 and Bid also leading to an amplification of the intracellular death signal [47-49].

Although TRAIL induces apoptosis when given alone, it has been shown that combination of TRAIL with cytotoxic drugs as 5-fluorouracil, etoposide, paclitaxel, actinomycin C and cisplatin has an even higher apoptotic efficacy [50-59,6].

Whereas abundant data therefore support the combination of TRAIL with cytotoxic drugs, only limited studies of TRAIL combined with ionising radiation have been performed [31,33,60-62]. Except for breast and renal cancer, no data supporting the application of TRAIL in the field of radiation oncology are available. Up to now, statistical analysis of a synergistic efficacy have been presented rarely. In addition, potentially harmful effects of a combination on normal cells have not sufficiently ruled out.

## Methods

### Chemicals

All biochemicals were obtained from Sigma-Aldrich chemicals (Deisenhofen, Germany) unless otherwise specified. Hoechst 33342 was purchased from Calbiochem and dissolved in distilled water as 1.5 mM stock solution.

### Cell culture

The tumour cell lines MDA-MB 231, HCT-15, Colo 205, NCI H460, FaDu DD and SCC-4 cells were purchased from ATCC (Bethesda, MD, USA). HSF6/ HSF7 fibroblasts, HUVEC and SMC were kindly provided from H-P. Rodemann and R. Kehlbach (Tübingen, Germany). Respectively. All cells were grown in RPMI 1640 medium (Gibco Life Technologies, Eggenstein, Germany) and maintained in a humidified incubator at 37°C and 5% CO_2_. Normal human epithelial cells (HMEC, PrEC, RPTEC, SAEC) and hepatocytes were obtained from Clonetics/Cambrex (Taufkirchen, Germany). Cell culture was performed according to the manufacturer's protocols.

### TRAIL stimulation

TRAIL induced apoptosis was induced with recombinant human TRAIL/TNFSF10 (R&D Systems, Wiesbaden-Nordenstadt, Germany) in concomitant or sequential application with irradiation.

### Irradiation

Cells were irradiated with 6 MV Photons using a Siemens Mevatron linear accelerator with a dose rate of 4 Gy per min at room temperature.

### Quantification of apoptosis induction

Apoptosis induction was quantified by counting of cells with a characteristic apoptotic morphology after DNA staining with Hoechst 33342. Cells were stained by incubation with Hoechst 33342 at a final concentration of 1.5 μM for 15 min. Microscopy was performed using a Zeiss Axiovert 200 microscope (Carl Zeiss, Jena, Germany) using an excitation wavelength filter of 380 nm. All apoptotic rates were means of at least three independent experiments. The given error bars represent the standard error of the mean from independent measurements of the same cell batch.

### Westernblotting

Cells (1 × 10^6^) were lysed for 30 min in a lysis buffer containing 25 mM HEPES, 0.1% SDS, 0.5% deoxycholate, 1% Triton X-100, 10 mM EDTA, 10 mM NaF and 125 mM NaCl on ice. After removing insoluble material by centrifugation for 10 min at 12.000 g, 20 μg lysate was separated by SDS-PAGE. Blotting was performed employing a tank blotting apparatus (Biorad, Munich, Germany) onto Hybond C membranes (Amersham, Braunschweig, Germany). Equal protein loading was confirmed by Ponceau S staining (Sigma). Blots were blocked in PBS buffer containing 0.05 % Tween 20 and 5% bovine serum albumin at 4°C over night. Primary antibodies were detected after repeated washings with PBS/Tween 20 (0.05%) of the membrane, using a secondary antibody (anti IgG-AP 1:10.000, Santa-Cruz-Biotech, Heidelberg, Germany) diluted in PBS/Tween and incubated for 3 hours at room temperature and washed three times with PBS/Tween. Detection of antibody binding was performed employing enhanced chemoluminescence (CSPD^®^-Solution Tropix, Applied Biosystems, MA, USA). PARP cleavage was tested using a polyclonal antibodies for cleaved and uncleaved PARP from Boehringer (Mannheim, Germany) in a 1: 1000 dilution. Monoclonal antibodies for caspase 8 were a gently gift from Prof. K. Schultze-Osthoff and used in a 1:45 dilution. β-Actin (Santa Cruz, Heidelberg, Germany) antibody was used in a 1: 5000 dilution.

### Receptor expression

Cells (0,2 × 10^6^) were washed twice with PBS and incubated for 30 min with PE-labeled anti-R1/DR4 or -R2/DR5-antibody (R&D Systems, Wiesbaden-Nordenstadt, Germany) at a dilution of 1: 400 in 0,5% FCS/PBS. FACS analyses of superficial receptor expression was performed according to manufacturer's protocol with the Quantibrite™ kit from BD (Heidelberg, Germany).

### Statistical analysis

Efficacy of the combined modalities were evaluated by the isobolic method [63].

## Results

### Ionising radiation sensitises solid tumour cells to TRAIL induced apoptosis

Rates of apoptosis induction in response to ionising radiation or TRAIL alone and after combination were determined. Since previous studies on Jurkat T cells or breast cancer cells demonstrated an upregulation of TRAIL receptor R2/DR5 after combined treatment with TRAIL and irradiation [54,31] two different application schedules were tested. TRAIL was either applied directly after cell irradiation or 12 hours later, to allow for receptor upregulation.

**Figure 1:**
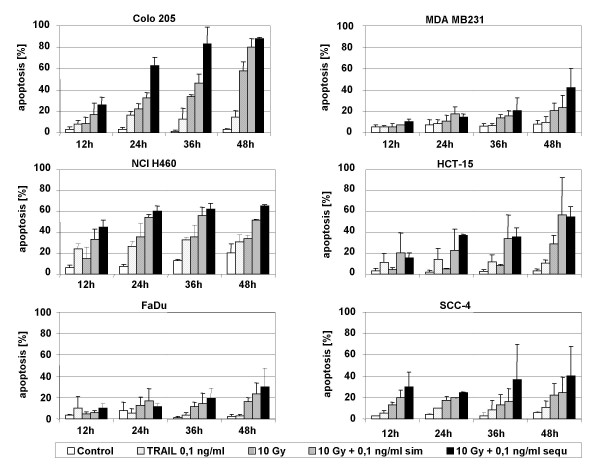
**Time course of induction of apoptosis in six solid tumour cell lines. **Apoptosis was determined by microscopic evaluation of Hoechst stained cell nuclei 12 to 48 h after treatment with TRAIL 0,1 ng/ml and 10 Gy alone, and after simultaneous and sequential application of combined therapy. Data represent means of three independent experiments; bars ± SD

As shown in figure 1, ionising radiation alone (10 Gy) at 48 h induced apoptosis in all cell systems from e.g. 16,0 % in FaDu cells and 34,1% in NCI H460 up to 58,0 % in Colo 205 cells, whereas 0.1 ng/ml TRAIL had a very limited activity in FaDu (3,0%) and Colo 205 cells (14,5%) and up to 30,7 % in NCI H460 tumour cells. In contrast, combination of irradiation (10 Gy) with immediate TRAIL application (0,1 ng/ml) was associated with a much higher apoptotic response (e.g. FaDu 23,3 %, NCI H460 51,9% and Colo 205 80,3%). This effect was even more pronounced when TRAIL was applied 12 hours after irradiation (e.g. FaDu 30,0 %, NCI H460 65,2% and Colo 205 88,0%).

**Figure 2:**
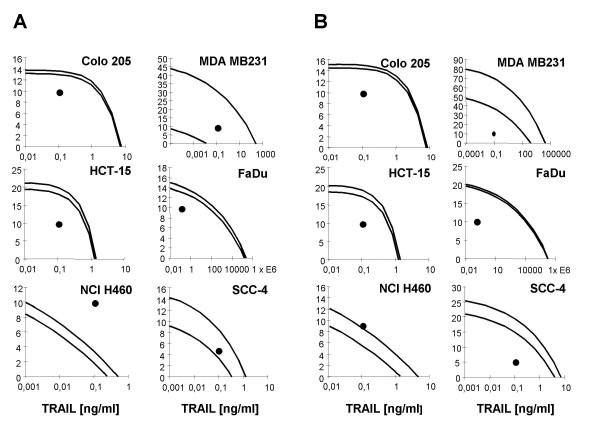
**Isobolographic analysis of apoptosis induction after combined treatment. **Apoptosis was determined by microscopic evaluation of Hoechst stained cell nuclei 48 h after combined treatment. Envelopes of additivity were calculated with data of 3 independently performed experiments. Datapoints below the curves resemble a synergistic effect, datapoints between the curves demonstrate an additive effect and above the curves a subadditive response of combined treatment. A : tumour cells were treated simultaneously with 10 Gy and 0,1 ng/ml TRAIL. B: cells were irradiated with 10 Gy 12 h prior to treatment with 0,1 ng/ml TRAIL.

In order to test whether the effect of TRAIL and radiation was additive or synergistic an isobologram analysis was performed. Even when applied concomitantly the interaction was synergistic in some cell systems (figure 2a, Colo205, HCT 15 and FaDu) but additive in others (MDA MB 231 and SCC-4). When TRAIL was applied 12 hours after irradiation the interaction was synergistic in all but one cell system (figure 2b).

### Processing of caspase 8 and PARP

**Figure 3:**
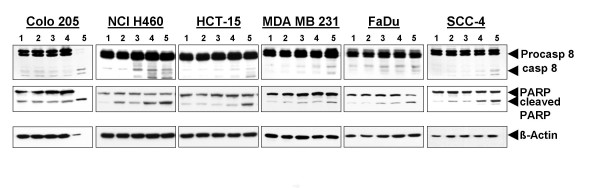
**Westernblot analysis of Caspase-8 activation and PARP-cleavage. **Lysates were prepared as described in methods. In six tumour cell lines(Colo 205, NCI H460,, HCT-15, MDA MB 231, FaDu and SCC-4) caspase-8 and PARP cleavage was analysed in untreated cells (lane 1), 24 h after treatment with TRAIL 0,1 ng/ml (lane 2) and 10 Gy alone (lane 3), after simultaneous (lane 4) and sequential application (lane 4) of combined therapy. Every cell line shows a different cleavage pattern according to the rate of apoptosis induction. β-Actin staining was used as loading control. The mapped blots represent each one of three independently performed immunoblots.

In order to substantiate the findings on apoptosis induction, caspase activation was verified by analysis of caspase-8 and the processing of the caspase-3 substrate PARP 24 hours after TRAIL application. In keeping with the above results, the most prominent effects were found for Colo 205 and NCI H460 cells with strongly increased caspase-8 and PARP processing after combined treatment. Colo 205 cells were particularly sensitive to sequential application of irradiation and TRAIL, whereas less intensive caspase-8 and PARP processing was found for MDA MB231, SCC4 and FaDu (fig. 3). These data correlate well with the kinetics of apoptosis induction as determined by fluorescence microscopy of Hoechst stained cells (fig. 1).

### Irradiation induced regulation of TRAIL receptors

**Figure 4:**
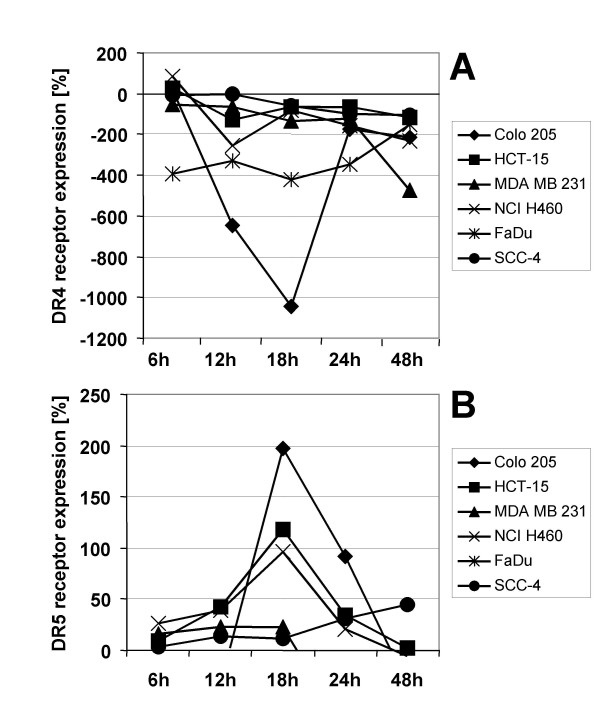
**Surface expression of TRAIL receptors after irradiation with 10 Gy in six tumour cell lines**. Quantification of receptor expression was performed 6 to 48 h after irradiation by FACS analyis using the Quantibrite™ evalution system from BD(Heidelberg, Germany) according to manufacturer's instructions. Data shown are from one representative experiment (n ≥ 3). A: Cell surface expression of R1/DR4 B: Cell surface expression R2/DR5

In order to analyse the role of TRAIL receptor regulation in combined therapy with irradiation and TRAIL receptor expression was quantified by flow cytometry using the Quantibrite™ kit system from Becton Dickinson (Heidelberg, Germany). No upregulation of DR4/R1 was found in all tested tumour cell lines. Instead, a subtle downregulation of DR4/R1 after irradiation was observed (fig. 4A). Fig. 4B demonstrates upregulation of TRAIL receptor DR5/R2 12–18 h after irradiation with 10 Gy in four of six tested cell lines. Colo 205 cells showed the most pronounced receptor upregulation of 196,8%. In HCT-15 and NCI H460 cells an upregulation of R2/DR5 of 118,0% resp. 96,4% could be measured. In MDA MB231 cells and SCC-4 cells no significant upregulation of receptors was found. FaDu cells do not express TRAIL-receptor R2/DR5 and therefore no upregulation of R2/DR5 could be observed after treatment.

### Combined treatment of TRAIL and ionising radiation do not damage normal tissue

**Figure 5:**
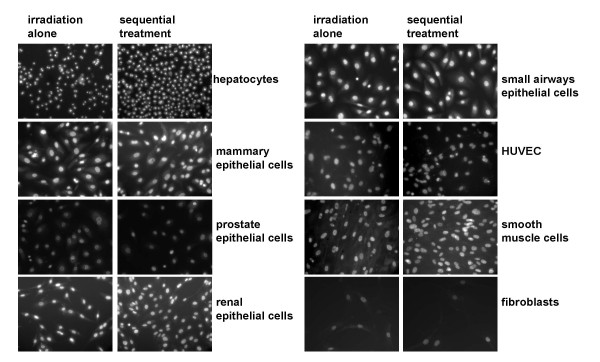
**Microscopic evaluation of normal tissue cells. **Lack of apoptosis was determined by microscopic evaluation of Hoechst stained cell nuclei. Micrographs depict cells of eight different normal tissues, 48 h after irradiation alone and after sequential treatment with 10 Gy 12 h previously to application of 1,0 ng/ml TRAIL.

As stated above, hardly any data on normal tissue toxicity after combined treatment are available. We therefore analysed the effect of irradiation plus TRAIL on human hepatocytes, fibroblasts (HSF6 and 7), epithelial cells from prostata (PrEC), kidney (RPTEC), breast (HMEC) and small airways (SAEC), endothelial cells from umbilical cord (HUVEC) and small muscle cells (SMC). Normal tissue cells were treated with 10 Gy and a tenfold higher dosage of TRAIL (1 ng/ml) as used for tumour cells. For evaluation of apoptosis strict morphologic criteria as condensation of chromatin and nuclear fragmentation were used. 48 h after combined treatment no relevant sensitisation of normal tissue cells to TRAIL induced apoptosis could be detected in all cultures. Moreover, even preirradiation did not sensitise normal cells to TRAIL induced apoptosis as observed in tumour cells (table 1 and fig. 5).

## Discussion

Based on the rationale that radiation and TRAIL induce cell death via distinct but overlapping cell death pathways, tumour cell lines and normal tissue cultures were subjected to either radiation or TRAIL alone or combined with varying application schedules. Our data show that combining radiation with TRAIL induces apoptosis in a significantly higher percentage than either treatment alone. It is important to note that for TRAIL stimulation in our experiments on tumour cells very low concentrations of TRAIL were used (0.1 ng/ml). The pharmacodynamic properties of TRAIL, especially the peak plasma levels in humans, are not known. Since it is likely that toxicity rises with higher doses of TRAIL, the observation of pronounced effects on tumour cell kill at such low doses is particularly intriguing.

Any combination of radiation with TRAIL proved to be more effective than either one alone; however depending on dose level and schedule of stimulation less than additive, additive and synergistic effects were detectable. When TRAIL was applied simultaneously with irradiation three of the six cell systems reacted synergistically. In contrast, five of the six cell systems reacted with synergistic effects when TRAIL was given sequentially 12 hours after irradiation. One of the cell lines displayed only less than additive effects.

Statistical analysis confirmed, that synergistic effects are more pronounced and occur with greater likelihood after sequential application of TRAIL after irradiation when compared to concomitant treatment schedules.

Possible explanations for the positive interaction of TRAIL and DNA damaging agents including ionising radiation are being disputed. Since the sensitisation was associated with upregulation of the DR5 receptor in some experimental settings [31,54,64,68-70], it is thought that the synergy is based on the increased surface density of the death receptors. Our data support the notion that DNA damage leads to an increased surface expression of the DR5 receptor. However, no tight correlation between receptor upregulation and magnitude of cell kill was observed. It has been suggested, that intact p53 is essential for upregulation of R2/DR5 death receptor expression by ionising radiation [64,68]. However, at least in one of our cell lines (HCT-15) known to harbour a non-function p53 DR5 upregulation was clearly upregulated. Thus, alternative p53 independent pathways for the upregulation of DR5 may exist. This finding is in accordance with data on p53 ^-/- ^HCT-116 cells and mouse embryonic fibroblasts showing that NF-κB may also be important for a irradiation induced upregulation of death receptors [65].

Recently, it was shown in overexpression experiments of NF-κB that its subunits c-Rel and RelA regulate expression of cell death molecules in a differential manner. This suggests that RelA, in contrast to c-Rel, acts as a survival factor by inhibiting expression of DR4/DR5 and caspase-8 and up-regulating cIAP1 and cIAP2 [71]. This process depends also on cell type and microenvironment [54,65]. Therefore NF-κB subunits seem to play an ambiguous role in regulation of apoptotic pathways. To know its exact role in radiation induced cell death further research is necessary. Additionally, conflicting data regarding the role of Bcl-2 in death receptor-mediated apoptosis have been provided in the past few years. Interestingly, new data point to a complex relationship between Bcl-2-mediated inhibition of apoptosis and the Bcl-2 protein expression level, the strength and the duration of the death receptor stimulus [66]. Therefore, Bcl-2 expression levels might play a critical role in the modulation of TRAIL sensitivity of tumour cells.

Recently, it has been shown that the interaction of 5-FU with TRAIL is strictly Bax but not Bak dependent in HCT 116 cells [67]. Thus, these experiments suggest also a critical role for the proapoptotic Bcl-2 homolog Bax in linking the TRAIL death receptor pathway to the mitochondrial apoptosis signalling cascade.

However, the general mechanism of the positive interaction of TRAIL with irradiation remains unclear. It may ultimately turn out, that manifold mechanisms exist and only some mechanisms will be operative in a single cell system [68].

The second part of our experiments the toxicity of TRAIL combined with radiation on normal tissues. In order to be able to draw relevant conclusions for normal tissue cells [8], 10 fold higher doses of TRAIL were used in these experiments. The first important finding is that TRAIL did not induce apoptosis in any of our cell systems including human liver cells. Thus, our experiments confirm the high tumour cell specificity of TRAIL [6,7]. Prior to embarking upon a phase I trial combining TRAIL with irradiation we wished to show a lack of sensitisation to TRAIL by pre-irradiation in normal tissues, compared to tumour cells. Using a wide array of normal cell systems we could not detect any effects of a pre-irradiation on TRAIL sensitivity. Combination of TRAIL with irradiation showed no increase in toxicity over radiation alone. These results are in good accordance with data from Shankar and coworkers showing that TRAIL induced apoptosis rates are only mildly enhanced by a preirradiation of non-malignant human prostate epithelial cells [68].

## Conclusions

Our data suggest that TRAIL has great potential in cancer treatment, especially in sequential combination with radiotherapy. We did not observe any sensitising effect of the sequential treatment on TRAIL sensitivity in normal tissue cells. Xenograft experiments designed to answer the questions regarding the short and long term efficacy of a combination of radiation with either TRAIL or TRAIL specific antibodies are underway in our laboratory.

## Abbreviations

APAF, apoptosis protease activating factor; FADD, Fas-associated death domain protein; NFκB, nuclear factor κB; PARP, poly-ADP-ribosyl polymerase; TNF, tumor necrosis factor; TRAIL, TNF-related apoptosis inducing ligand. HMEC: human mammary epithelial cells, PrEC: human prostate epithelial cells, RPTEC: epithelial cells of renal proximal tubule, SAEC: small airways epithelial cells, HUVEC: human epithelial cells of umbilical vein, SMC: smooth muscle cells, HSF 6,7: human fibroblasts

## Competing interests

The author(s) declare that they have no competing interests.

## Authors' contributions

PM performed FACS-analysis and microscopic evaluation of Hoechst stained cells, analyzed the data, participated in the conception of the trial and participated in the preparation of the manuscript. AS participated in microscopic evaluation of tumour cell apoptosis and accomplished analysis of the resulting data. VJ participated in receptor analysis and microscopic evaluation of Hoechst stained cells. HF carried out Western blotting. PTD participated on the preparation of the manuscript. WB performed isobologramm analysis. CB participated in the conception, design of the study, coordination of the study as well as preparation of the manuscript. All authors read and approved the final manuscript.

## Pre-publication history

The pre-publication history for this paper can be accessed here:



## Figures and Tables

**Table 1:** Normal tissue toxicity.

	**Hepatocytes**	**HMEC**	**PrEC**	**RPTEC**	**SAEC**	**HUVEC**	**SMC**	**HSF6**	**HSF7**
**Control**	0,3 ± 0,5	0,3 ± 0,5	0,3 ± 0,5	0	0,3 ± 0,5	0,2 ± 0,2	0,5 ± 0,2	0	0

**TRAIL 1,0 ng/ml**	0,3 ± 0,5	0,5 ± 0,4	0	0	0	0,2 ± 0,1	0,1 ± 0,1	0	0

**10 Gy**	0	0,5 ± 0,5	0	0	0,1 ± 0,2	0,3 ± 0,2	1,5 ± 0,5	0	0

**Simultaneous combined therapy**	0	1,6 ± 0,5	0,7 ± 0,5	0	0	0,3 ± 0,1	1,3 ± 0,9	0	0

**Sequential combined Therapy**	0,3 ± 0,5	0,8 ± 0,5	1,5 ± 1,0	0	0,3 ± 0,5	0,2 ± 0,2	1,2 ± 1,0	0	0

Cells were treated with 1,0 ng/ml TRAIL, 10 Gy, simultaneous combined therapy and with 10 Gy 12 h prior to TRAIL application. Microscopic evaluation of Hoechst stained cell nuclei with strict apoptotic criteria as chromatin condensation and nuclear fragmentation was performed 48 hours after treatment. Table shows percentage of apoptotic cells ± SD (n = 3).
